# Exposure to environmental occupational constraints and all-cause mortality: Results for men and women from a 20-year follow-up prospective cohort, the VISAT study. Be aware of shift-night workers!

**DOI:** 10.3389/fpubh.2022.1014517

**Published:** 2022-11-10

**Authors:** Yolande Esquirol, Samantha Huo Yung Kai, Camille Carles, Jean-Claude Marquié, Audrey Fernandez, Vanina Bongard, Jean Ferrières

**Affiliations:** ^1^UMR 1295, Centre d'Epidémiologie et de Recherche en santé des Populations, Université Paul Sabatier Toulouse III – Inserm, Toulouse, France; ^2^Occupational Health Department, CHU-Toulouse, Toulouse, France; ^3^Department of Epidemiology, CHU de Toulouse, Toulouse, France; ^4^Occupational Health Department, Equipe EPICENE, CHU de Bordeaux, University Bordeaux, Inserm UMR 1219, Bordeaux, France; ^5^Cognition, Langues, Langage, Ergonomie, Centre national de la recherche scientifique, Université Toulouse 2 Jean Jaures, University of Toulouse, Toulouse, France; ^6^UMR 1295, Centre d'Epidémiologie et de Recherche en santé des POPulations, Université Paul Sabatier Toulouse III–Inserm, Toulouse, France; ^7^Epidemiology Department CHU de Toulouse, UMR 1295, Centre d'Epidémiologie et de Recherche en santé des POPulations, Université Paul Sabatier Toulouse III-Inserm, Toulouse, France; ^8^Department of Cardiology, CHU de Toulouse, UMR 1295, Centre d'Epidémiologie et de Recherche en santé des POPulations, Université Paul Sabatier Toulouse III–Inserm, Toulouse, France

**Keywords:** mortality, occupational exposed workers, organizational factors, occupational physical activity, shift work, work environment, psychological factor

## Abstract

**Objective:**

To determine the predictive value of the large panel of occupational constraints (OC) on all-cause mortality with a 20-year follow-up, in general population of workers.

**Methods:**

In VISAT prospective cohort study, 3,138 workers (1,605 men; 1,533 women) were recruited during the periodic work health visits conducted by occupational physicians. OC (physical, organizational, psychological and employment categories) were collected through self-questionnaires. Exposure durations of each OC were divided by tertile distribution. Cox-regression models were performed to analyze the associations between all-cause mortality and each OC first separately and simultaneously in a single model.

**Results:**

The mortality rates were higher among exposed participants to most of OC compared to those unexposed. Being exposed and longer exposure increased the risks of all-cause mortality for exposures to carrying heavy loads, loud noise, working more than 48 h/week, starting its first job before 18 years old although these risks became non-significant after adjustments for cardiovascular risk factors. Shift work and night work confirmed a high risk of mortality whatever the adjustments and notably when the other occupational exposures were taking into account, with, respectively: HR: 1.38 (1.01–1.91) and 1.44 (1.06–1.95). After adjustments being exposed more than 13 years to a work requiring getting-up before 5:00 a.m. and more than 16 years in rotating shift work significantly increased the risk of mortality by one and a half.

**Conclusion:**

The links between each OC and all-cause mortality and the role of individual factors were stressed. For night-shift workers, it is urgent to implement preventive strategies at the workplace.

## Introduction

Around the world, substantial inequalities persist in all-cause mortality, with a clear disparity between occupational-social categories. By the early 1980's, results obtained from the Whitehall study have reported on a higher all-cause mortality rate among blue collars compared to white collars (1). Since then, these findings have been confirmed (2) in several countries, regardless the type of social system (3). France is no exception to this observation (4). The lifestyle behaviors and occupational environment have often been proposed to explain such inequalities (2).

However, the major studies undertaken to explain mortality mostly used a list of occupations and social status in specific populations without consistent adjustments and their conclusions have also been criticized (5). The list of occupations does not always reflect the type of occupational constraints (OC) imposed on workers and is of little help to determine preventive strategies to improve the occupational environment.

Few studies have been specifically undertaken to analyze the potential impact of some specific OC on all-cause mortality. Working more than 55 h/week has been associated with an increased risk of all-cause mortality but only among men carrying out a routine occupation (6). Few studies have focused on the specific impact of a high level of occupational physical activity on all-cause mortality, while it has been suggested as a risk factor specifically for cardiovascular mortality (7–9). Also, the impact of shift work on all-cause mortality is still debated in meta-analyses including few prospective studies with inconsistent definition of working hour pattern (10, 11). Despite the predicted value of stress on morbidity and on cardiovascular mortality (12), few studies have analyzed the effect of stressful working constraints on all-cause mortality.

Therefore, little evidence is given by the existing literature to suggest the potential deleterious impact of OC on mortality. The main confounders were not systematically considered and the results were established most often in men and rarely in women, while the differences in mortality rates remains between men and women (13).

Moreover, we can assume that exposure duration to these OC could differently impact all-cause mortality. The knowledge of these thresholds could induce some management changes of working organization in companies.

Moreover, few studies have considered the large diversity of combined OC to which workers could be exposed during their working periods. Thus, conclusion concerning potential effects of OC on all-cause mortality remains difficult to establish and a wider understanding of involved occupational factors is needed. The purpose of the present work was to analyze the value of a large panel of OC and the exposure duration to predict all-cause mortality, after a median follow-up period of 20 years from the VISAT cohort study, with adjustment for individual risk factors, in particular cardiovascular risk factors, as well as taking into account occupational co-exposures.

## Materials and methods

### Study design

The present analysis is based on data from the VISAT prospective cohort. The VISAT study started in 1996 and was designed to understand relationships between work conditions, age and health. Volunteer working or recently unemployed men and women aged 32, 42, 52, and 62 years at the initial collection (1996) were recruited in Southwestern of France with 20-year mortality follow-up.The workers were employed in a wide range of occupational sectors (32.4% from manufacturing industries, 27.9% from health and social work, 7.5 % from public administration, 7.9% real estate, rental, business services, 5.2 % from transports, 4.2 from financial activities, 1.7% from construction, 2.3% from community and social and personal services).

Data were collected at baseline through self-administered questionnaires, medical questionnaires and clinical examinations conducted by trained occupational physicians during the mandatory periodic work health visits. Further detailed information on the VISAT study protocol has been previously described elsewhere (14). VISAT study is registered in “Le Portail Epidemiologie – France- Aviesan –Id 3666”

### Ethics approval

Participants were informed and volunteered to participate in the VISAT study. Informed consent was sought and granted. The VISAT project obtained agreement from the French National Committee on Computer Files and Civil Liberties (CNIL). All procedures followed international standards pertaining on human research in accordance with the Declaration of Helsinki. Acceptance by the CNIL was renewed in 2009 for the additional use of mortality data (CNIL 09.149). This observational study follows the Strobe guidelines.

### Assessment of occupational constraints

Four categories of OC were analyzed (physical constraints; organizational constraints; psychological constraints; employment characteristics).

Each occupational exposure was coded as: past or current exposure (Yes) vs never exposed (No). Exposure duration of each OC was divided into four classes: The exposure duration of each OC assessed by self-questionnaire at initial recruitment to characterize previous exposure duration was divided into 4 classes: never exposed during working periods (no)/exposure duration classified by tertile distribution.

1) Physical constraints included the following tasks: carrying heavy loads or similarly intense physical effort, being exposed to loud noise (preventing from hearing a person located 2 or 3 meters away even if talking loudly).

2) Concerning organizational constraints and exposure durations, two types were analyzed: (i) information on the weekly working hour duration (working more than 48 h/week (ii) working hour pattern (four variables): working in rotating shift; night work (having an occupation which prevented from sleeping during the night at least 50 nights/year); working after midnight (bedtime after midnight at least 50 nights /year because of work); working before 5:00 a. m. (getting-up before 5:00 a.m. at least 50 mornings/year because of work).

Moreover, a new variable was constructed with three classes: day workers/shift work without night work/ night work at least 50 work nights per year with or without shift work. Thus, day workers were defined as workers who never had a work schedule involving a rotating shift work nor an occupation which prevented them from sleeping during the night for more than 50 nights/year at the initial data collection or in the past.

Shift workers without night work had rotating shift work at the initial data collection or in the past but did not have work which prevented them from sleeping during the night for more than 50 days/year.

Night workers were participants who had an occupation which prevented them from sleeping during the night at least than 50 days/year, with or without rotating shift work at the initial data collection or in the past.

These definitions were used in several prior large French studies: ESTEV (15), SUMMER (16) or previous VISAT publications (17), and also as a national classification of night work in other countries (i.e., Germany, Austria) (18).

3) Questions used to define psychological constraints were similar to those used in the European survey on working conditions (19) and also to those previously applied in the ESTEV and VISAT studies (20–22). The questions included in the self-questionnaire allowed the assessment of both psychological dimensions proposed by the Karasek model (decision latitude and psychological demand) (23). By combining the levels (high or low) of each dimension, four different categories of job strain were defined: “low job strain” (low psychological demand and high decision latitude); “passive job” (low psychological demand and low decision latitude); “active work” (high psychological demand and high decision latitude); “high job strain” (high psychological demand and low decision latitude). Finally, job strain was analyzed by using two categories: exposed to low or passive job strain vs. exposed to active or high job strain. No data concerning exposure duration of this occupational constraint was available in this study.

4) Job-related risk factors were included to better describe some characteristics of employment: age at first job, dichotomized into younger than 18 or 18 years and older; according to the national classification of profession and occupational social categories of the National Institute of Statistics and Economic, occupational social status was regrouped into 3 categories: white collars/employees/manual workers.

### Assessment of individual risk factors

In order to take into account potential confounding factors known to interact with all-cause mortality, the following variables were collected: age at baseline (32 and 42 [this class were aggregated to avoid categories with low mortality rates]/52/62 years-old), gender, educational level ( ≤ A-level vs. > A-level), current tobacco consumption (No/Yes), body mass index (BMI > 25 kg/m^2^: No/Yes). At baseline automatic arterial blood pressure was measured three times, in the presence of a practitioner. Participants with Systolic Blood Pressure (SBP) ≥ 140 mmHg and/or Diastolic Blood Pressure (DBP) ≥ 90 mmHg or exhibiting an antihypertensive treatment constituted the hypertension group (No/Yes).

Medical history of diabetes and history of another disease (cancer, hematopoietic, endocrine, neurological, ophthalmological, otorhinolaryngological, cardiovascular, pulmonary, digestive, skin, musculoskeletal, gynecological, urological diseases and congenital defects), were dichotomized (No/ Yes).

### Mortality

Vital status was obtained from the French National Registry of People Identification. Dates of death were collected from French Epidemiology Center for medical causes of death and validated by an expert's committee. After a median follow-up period of 20 years (December 2016), 242 events were recorded.

### Statistical analysis

In the whole sample, descriptive analyses were conducted at baseline for individual and OC (exposed or not exposed, and exposure duration). The occupational exposure duration was expressed in years, categorized by tertiles; for each tertile, minimum, maximum and median values were provided (no exposure constituted the reference class). Associations with mortality status were determined by *p*-value for Pearson's chi2 χ2 test. The analyses have been carried out in whole sample. Mortality rates per 1,000 person-year and 95% confidence interval were calculated. Kaplan Meier survival analyses according for exposure duration of some OC in whole sample were provided. The log-rank test was used to test whether the difference between survival times between the groups was statistically different or not, but did not allow to test the effect of the other independent variables.

Cox proportional hazard regression analyses were undertaken to determine the impact of each OC and the exposure duration of each of them on all-cause mortality in non-adjusted and adjusted models (age, gender, diabetes history, hypertension, overweight, tobacco habits, occupational social categories, other disease history). As social variable, all the analyses were also carried out with educational level instead of occupational social categories: no substantial difference of the results were observed (not shown).

Finally, all OC linked to mortality in bivariate analyses (*p*-value < 0.20) were considered together in a single model as potential explanatory variables in adjusted multivariate cox-models. Backward procedures were applied to determine which OC were significantly (*p* < 0.05) and independently associated with all-cause mortality. A likelihood-ratio test was applied at each step of the backward procedure, before removing a variable from the model in adjusted model. Interactions in the initial model (notably between the occupational constraints) and in the final model were checked.

For all the analyses, the probability of dying during 20-year follow-up was expressed by hazard risk (HR) and 95% confidence interval. Proportional-hazard assumption was tested using Schoenfeld residuals for each covariate. Tests of time-varying effect of covariates were applied (no time interaction was found except for the variable ‘duration of exposure to carrying and lifting heavy loads'). Interactions between variables of interest were checked; generalized variance inflation was used to confirm absence of collinearity; significance of *p*-value was set at 0.05

All statistical analyses were done using the Stata**^®^** software StataCorp. 2013. *Stata Statistical Software: Release 13*. College Station, TX: StataCorp LP.

## Results

A total of 3,237 volunteers participated in the VISAT study. Due to missing data for variables of interest, 99 (3.0%) people were excluded from the present analyses, resulting in a study population of 3,138 participants. For individual and occupational factors, no differences were found between included and drop-out participants (not shown).

The study population comprised 1,605 (51.1%) men, and included 14.5% of people aged 62 at baseline, 27.6% aged 52, and 57.9% aged 32 or 42 years.

Supplementary Table A describe the results of bivariate analyses and mortality rates according to baseline individual characteristics for the whole sample. Two hundred and forty-two deaths were recorded after 20-years of follow-up (7.7 % of all participants; 4.3% of women and 10.9% of men) corresponding to an all-cause mortality rates of 3.9 (95% IC: 3.4–4.4) per 1,000 person-years for whole sample and of 5.6 (95% IC: 4.8–6.4) and 2.1 (95% IC: 1.7–2.7) for men and women, respectively.

As expected, elevated all-cause mortality rates were observed among older participants, subjects with hypertension, tobacco consumers, overweight participants and those with a medical history of diabetes at baseline (Supplementary Table A).

Tables 1–3 present the mortality rates and the results of the proportional cox-analyses undertaken to analyze the associations between each OC and all-cause mortality in non-adjusted and adjusted models for individual factors, in the whole sample. HR (95% CI) were provided for each category of OC which were classified into four types: physical constraints (carrying or lifting heavy loads, loud noise), working hour schedule (working ≥ 48 h/week, shift –night work), psychological constraints, and employment characteristics.

**Table 1:** Occupational physical constraints and all-cause mortality.

**Occupational physical constraints**							
***N* = 3,138; death:** ***N* = 242 events**	**%**	**% deaths**	***p*-value**	**Person-time**	**Mortality Rate (95% CI)**	**Model A** **HR (95% CI)**	**Model B** **HR (95% CI)**
**Heavy lifting or carrying:**							
No	58.2	6.7	**0.01**	36,447	3.3 (2.8–3.9)	Ref	Ref
Yes	41.8	9.2		25,801	4.6 (3.9–5.6)	1.39 (1.08–1.79)	1.26 (0.97–1.65)
**Exposure duration, years (ref: no exposure)**							
1^th^: > 0–8, 4	15.6	6.7	**0.000**	9,685	3.4 (2.4–4.8)	1.02 (0.69–1.49)	1.12 (0.75–1.65)
2^th^: > 8–18, 12	13.5	8.2		8,419	4.1 (2.9–5.8)	1.24 (0.85–1.81)	1.26 (0.85–1.85)
3^th^: > 18–38, 25	12.7	13.3		7,695	6.8 (5.2-8.9)	**2.03 (1.47-2.81)**	1.40 (0.99-1.98)
P for trend						**0.000**	**0.04**
**Loud noise**							
No	73.9	6.5	**0.000**	46,198	3.3 (2.8–3.8)	Ref	Ref
Yes	26.1	11.1		16,050	5.7 (4.6–6.9)	**1.74 (1.34**–**2.26)**	**1.35 (1.03**–**1.77)**
**Exposure duration, years (ref: no exposure)**							
1^th^: > 0–6, 4	8.9	7.2	**0.000**	5,534	3.6 (2.3–5.6)	1.11 (0.69–1.77)	0.94 (0.59–1.51)
2^th^: > 6–16, 10	8.7	10.9		5,332	5.6 (3.9–8.1)	**1.73 (1.17**–**2.56)**	1.36 (0.91–2.03)
3^th^: > 16–38, 24	8.5	1.3		5,183	7.9 (5.8–10.7)	**2.45 (1.74**–**3.46)**	1.31 (0.91–1.90)
P for trend						**0.000**	0.08

Mortality rate /1,000 person-year and CI; *p*-value for Pearson's chi2 testing associations between definite variables and all-cause mortality; Occupational exposure duration is expressed in years, categorized by tertiles; for each tertile, minimum, maximum, and median values are provided. Cox analyses provided: HR: hazard risk; 95% IC: 95% confidence interval; Model A: unadjusted model; Model B: adjusted model on age, gender, diabetes history, hypertension, overweight, tobacco habits, occupational social categories, other disease history. Interactions between variables of interest were checked; generalized variance inflation was used to confirm absence of collinearity; Proportional-hazard assumption was tested using Schoenfeld residuals for each covariate. Time-varying tests were applied. Significance of *p*-value was set at 0.05.

**Table 2:** Occupational organizational constraints and all-cause mortality.

**Occupational organizational constraints**							
***N* = 3,138; death: *N* = 242 events**	**%**	**% deaths**	***p*–value**	**Person–time**	**Mortality rate (95% CI)**	**Model A** **HR (95% CI)**	**Model B** **HR (95% CI)**
**Working** **≥48 h/week**,							
No	60.1	5.7	**0.002**	37,733	2.8 (2.3–3.4)	Ref	Ref
Yes	39.9	10.8		24,514	5.5 (4.6–6.5)	**1.95 (1.51–2.51)**	1.17 (0.89–1.53)
**Exposure duration, years (ref: no exposure)**							
1th > 0–5, 2	13.7	7.2	**0.000**	8,503	3.6 (2.6–5.1)	1.11 (0.69–1.77)	1.15 (0.59–1.51)
2^th^: > 5 −12, 8	14.3	9.8		8,776	5.1 (3.7–6.7)	**1.73 (1.17–2.56)**	1.05 (0.91–2.03)
3^th^: > 12–37, 20	11.9	16.0		7,235	8.2 (6.4–10.7)	**2.45 (1.74–3.46)**	1.31 (0.91–1.90)
P for trend						**0.000**	0.18
**Working hour schedule**							
Day work	60.3	6.5	**0.002**	37,721	3.3 (2.7–3.9)	Ref	Ref
Shift – no night work	20.9	8.2		13,000	4.1 (3.2–5.4)	1.26 (0.92–1.73)	**1.39 (1.01–1.92)**
Night work +/– shift work	18.8	10.9		11,537	5.5 (4.3–7.1)	**1.69 (1.25–2.29)**	**1.44 (1.06–1.96)**
**Rotating shift work** (exposure duration, years)							
No exposure	63.5	7.1	**0.035**	39,589	3.6 (3.1–4.2)	Ref	Ref
1^th^: > 0–6, 4	13.9	7.1		8,715	3.5 (2.5–5.1)	0.99 (0.67–1.46)	1.05 (0.7–1.6)
2^th^: > 6 −16, 11	11.7	7.9		7,260	3.9 (2.7–5.7)	1.11 (0.75–1.66)	1.25 (0.8–1.9)
3^th^: > 16–36, 22	10.9	11.6		6,683	5.9 (4.3–8.1)	**1.68 (1.18–2.38)**	**1.47 (1.1–2.1)**
**P for trend**						**0.01**	**0.03**
**Bedtime** **>** **midnight** (exposure duration, years)							
No exposure	79.4	6.9	**0.000**	49,561	3.5 (2.9–4.1)	Ref	Ref
1^th^: > 0–5, 2	7.3	7.4		4,553	3.7 (2.3–6.1)	1.07 (0.65–1.77)	1.07 (0.65–1.76)
2^th^: >5–12, 8	7.1	10.4		4,331	5.3 (3.5–7.9)	1.54 (0.99–2.37)	1.29 (0.83–2.00)
3^th^: >12–36, 20	6.2	15.2		3,802	7.8 (5.5–11.3)	**2.30 (1.56–3.39)**	1.30 (0.88–1.94)
P for trend						**0.000**	0.11
**Getting–up** **<** **5:00 AM** (exposure duration, years)							
No exposure	72.8	6.5	**0.000**	45,542	3.2 (2.7–3.8)	Ref	Ref
1^th^: > 0–4, 2	9.2	7.9		5,706	4.1 (2.6–6.1)	1.24 (0.80–1.92)	1.19 (0.76–1.86)
2^th^: >4–13, 8	9.1	9.8		5,620	4.9 (3.4–7.2)	**1.54 (1.03–2.30)**	1.14 (0.75–1.73)
3^th^: >13–36, 20	8.9	15.4		5,379	7.9 (5.9–10.7)	**2.49 (1.77–3.49)**	**1.54 (1.08–2.19)**
P for trend						**0.000**	**0.02**
**Working at night** (exposure duration, years)							
No exposure	81.4	6.9	0.004	50,721	3.5 (3.1–4.1)	Ref	Ref
1^th^: > 0–4, 2	6.6	10.1		4,081	5.1 (3.3–7.9)	1.47 (0.93–2.31)	1.52 (0.96–2.39)
2^th^: >4–12, 8	6.3	9.6		3,883	4.9 (3.1–7.6)	1.40 (0.87–2.25)	1.23 (0.76–1.98)
3^th^: >12–36, 20	5.7	13.6		3,398	7.1 (4.7–10.5)	**2.03 (1.33–3.11)**	1.27 (0.82–1.96)
P for trend						0.000	0.13

Mortality rate /1,000 person-year and CI; *p*-value for Pearson's chi2 testing associations between definite variables and all-cause mortality; Occupational exposure duration is expressed in years, categorized by tertiles; for each tertile, minimum, maximum and median values are provided. Cox analyses provided: HR: hazard risk; 95% IC: 95% confidence interval; Model A: unadjusted model; Model B: adjusted model on age, gender, diabetes history, hypertension, overweight, tobacco habits, occupational social categories, other disease history. Interactions between variables of interest were checked; generalized variance inflation was used to confirm absence of collinearity; Proportional-hazard assumption was tested using Schoenfeld residuals for each covariate. Time-varying tests were applied. Significance of *p*-value was set at 0.05.

**Table 3:** Occupational psychological and employment constraints and all-cause mortality.

**Occupational psychological and employment constraints**							
***N* = 3,138; death: *N* = 242 events**	**%**	**% deaths**	***p*-value**	**Person–time**	**Mortality rate (95% CI)**	**Model A** **HR (95% CI)**	**Model B** **HR (95% CI)**
**Job strain**							
low and passive	32.2	8.1	0.57	20,075	4.1 (3.3–5.1)	Ref	Ref
Active and high	67.8	7.5		42,172	3.8 (3.2–4.4)	0.93 (0.71–1.21)	1.05 (0.80–1.38)
**Employment**							
**First job** **<** **18 yo**,							
No	64.9	5.8	**0.000**	40,700	2.9 (2.4–3.5)	Ref	Ref
Yes	35.1	11.2		21,548	5.7 (4.8–6.8)	**1.96 (1.52–2.53)**	1.23 (0.93–1.64)
**Occupational social categories**							
White collars	40.5	8.1	**0.000**	25,151	4.1 (3.3–4.9)	Ref	Ref
Employees	35.4	5.3		22,255	2.6 (2.1–3.4)	0.65 (0.47–0.89)	0.79 (0.56–1.10)
Manual workers	24.1	10.6		14,842	5.4 (4.3–6.7)	1.32 (0.99–1.77)	1.10 (0.82–1.48)
P for trend						0.39	0.88

Mortality rate /1,000 person-year and CI; *p*-value for Pearson's chi2 testing associations between definite variables and all-cause mortality; Occupational exposure duration is expressed in years, categorized by tertiles; for each tertile, minimum, maximum and median values are provided. Cox analyses provided: HR: hazard risk; 95% IC: 95% confidence interval; Model A: unadjusted model; Model B: adjusted model on age, gender, diabetes history, hypertension, overweight, tobacco habits, occupational social categories, other disease history. interactions between variables of interest were checked; generalized variance inflation was used to confirm absence of collinearity; Proportional-hazard assumption was tested using Schoenfeld residuals for each covariate. Time-varying tests were applied. Significance of *p*-value was set at 0.05.

### Occupational physical constraints

The workers exposed to heavy lifting or carrying loads had a higher mortality rate compared to those unexposed. The risk of all-cause mortality increased with the exposure duration (P for trend 0.04). For workers exposed to more than 18 years of carrying heavy loads or similarly intense physical effort, the HR of all-cause mortality was increased by twice in unadjusted model and had risen by almost one-and-a-half after adjustments, even though a non-statistically significant risk was observed [HR: 1.40 (0.99–1.98)] (Table 1).

For the workers of the first class of exposure duration [(0–8) years], a time-varying effect was found. The HR for this class was evaluated at 2.92 (1.24–6.87) with 0.92 (0.86–0.98) for the time-varying covariate. Thus, the risk of all-cause mortality for workers exposed to carrying or lifting heavy loads was significantly increased at T0 and decrease by 8% per year during the 8 years of exposure duration.

For workers exposed to intense noise during working periods being exposed more than 6 years increased significantly the risk of all-cause mortality in unadjusted model, while the risk became statistical non-significant when confounding factors were taken into account (Table 1).

### Occupational organizational constraints

The occupational organizational constraints focused on the different working time patterns.

The mortality rate for people working more than 48 h/week was estimated at 5.5 (4.6–6.5). Regardless exposure durations and after adjustments, the risk of all-cause mortality was not significantly elevated for these workers 20 years later.

All-cause mortality risks increased with work requiring to have bedtime after midnight for work, and working at night in unadjusted model, while no significant risk was highlighted when adjustments for main confounding factors were applied. However, more the exposure duration of getting-up before 5:00 a.m.M increased, more the risk of all-cause mortality rose, and remained significant for the longest exposure duration after adjustment [exposure duration >13 years: HR: 1.54 (1.08–2.19)]. Moreover, working with rotating shift more than 16 years increased the risk of all-cause mortality by one and a half after adjustments: HR:1.47 (1.1–2.1). Compared to day workers, the workers with shiftwork without nights and those with night work (permanent night work or shiftwork) had significant increased risks of all-cause mortality after adjustments (Table 2).

### Occupational psychological and employment constraints

The job strain, resulting of the combination of decision latitude and psychological demand was divided in two classes: workers with low strain or a passive job compared to those with high strain or an active job. No significant difference of risk between the two classes was observed. No data concerning job strain exposure duration was available in this study (Table 3).

The participants who started their first job before 18 years old were represented by 42.6 % manual workers, 36.7 % employees and 20.7% white collars (*p* < 0.0001). The risk of all-cause mortality was significantly increased for people who began working before 18 years old, but remained non-significant after adjustments. Compared to white collars, the risks of all-cause mortality were not significantly elevated for employees and manual workers (Table 3).

**Figure 1:**
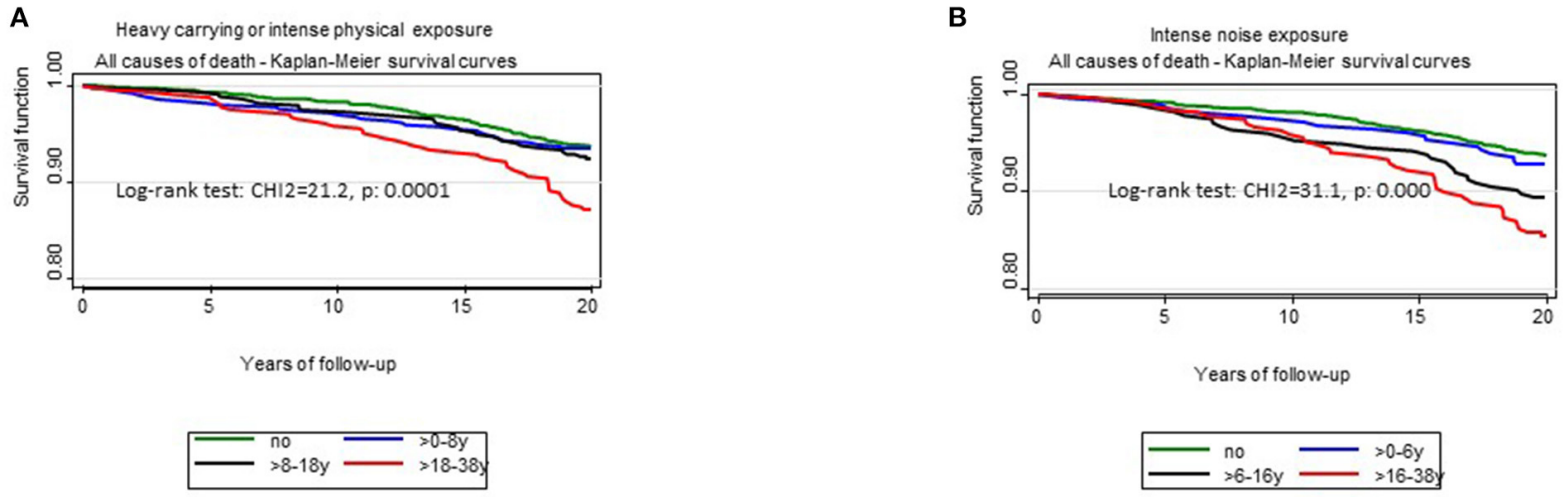
Kaplan-Meier survival curves for all-cause mortality according to exposure duration of occupational physical constraints. **(A)** Kaplan–Meier survival curves according to exposure duration to carrying heavy loads or similar intense physical activity at work. **(B)** Kaplan–Meier survival curves according to exposure duration to intense noise at work.

**Figure 2:**
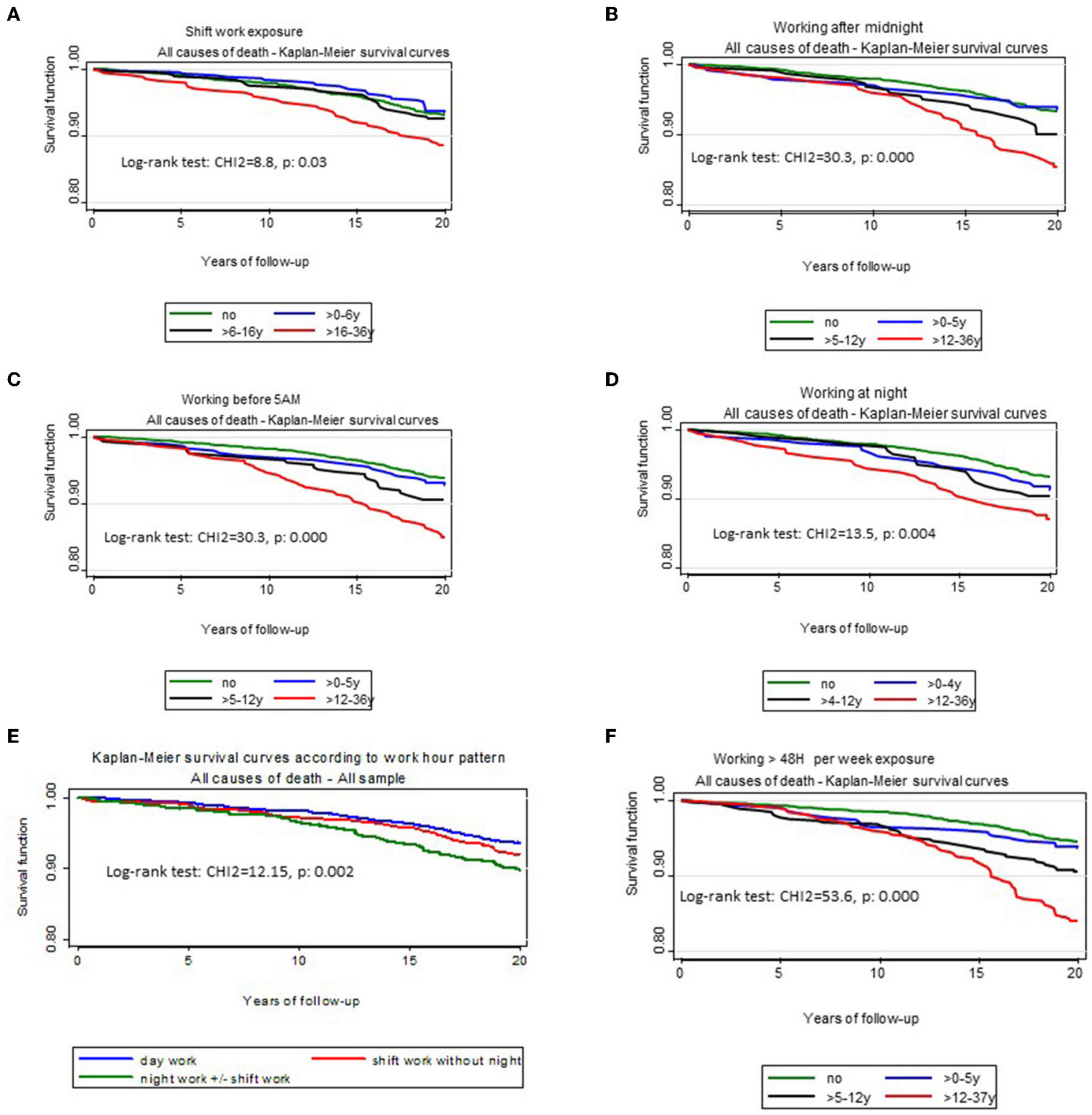
Kaplan-Meier survival curves for all-cause mortality according to exposure duration of occupational organizational constraints. **(A)** Kaplan–Meier survival curves according to exposure duration to shift work. **(B)** Kaplan–Meier survival curves according to exposure duration of working after midnight. **(C)** Kaplan–Meier survival curves according to exposure duration of working before 5:00 a.m. **(D)** Kaplan–Meier survival curves according to exposure duration of working at night. **(E)** Kaplan–Meier survival curves according to working hour schedule. **(F)** Kaplan–Meier survival curves according to exposure duration of working >48 h/week.

**Figure 3:**
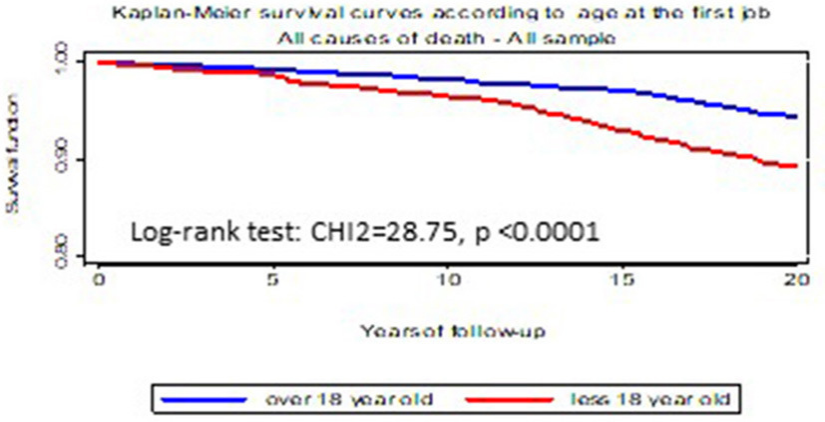
Kaplan–Meier survival curves according to age at first job.

Figures 1–3 illustrates Kaplan-Meier survival curves according to occupational exposures and exposure durations when people were exposed to lifting or carrying loads, loud noise, working hours ≥ 48 h/week, the different working hour patterns and Job strain The log rank tests showed significant differences according for the different exposure durations for most of physical and organizational constraints. Survival was higher among unexposed workers and among workers with a shorter exposure duration for each OC examined.

Finally, cox-analyses with backward procedures were conducted to explore the contribution of combined OC to all-cause mortality in full model with adjustments (Table 4).

**Table 4:** Contribution of individual factors and all combined occupational constraints to mortality in all population, after 20 years of follow-up: Backward cox-analyses.

**Occupational and individual factors**	**All** ***n*** = **3,138, death** ***n*** = **242**	
	**Adjusted HR**	**95% CI**
**Work schedule**		
Day work	**Ref**	
Shift work no night work	**1.38**	**(1.01 to 1.91)**
Night +/- shift	**1.44**	**(1.06 to 1.95)**
**Age**		
32–42 52 62	Ref 2.51 5.29	(1.79 to 3.50) (3.73 to 7.49)
**Hypertension**		
No Yes	Ref 1.58	(1.20 to 2.09)
**Smoking status**		
Never or former Current	Ref 1.94	(1.48 to 2.55)
**Diabetes**		
No Yes	Ref 1.66	(1.03 to 2.67)
**Gender**		
Men	Ref	
Women	0.51	(0.38 to 0.68)

Individual factors: gender, age, Hypertension, diabetes, BMI >25 Kg/m^2^, smoking status, other disease history; Occupational exposure: carrying heavy loads, intense noise, working >48 h/week; work schedule (day work vs shift and night work); job strain, age at first job; socio-professional categories; HR: hazard risk; 95% CI 95% confidence interval; No interaction and no collinearity in final models; Proportional-hazard assumption was tested using Schoenfeld residuals; no significant time-varying interactions of occupational covariates were found in final model. The interactions between occupational constraints in initial model were tested using Likelihood-ratio tests: non-significant.

Compared to day work, being exposed to shift work without night or to night work including or not shift work were the only OC that remained significantly and independently associated with all-cause mortality, with, respectively HRs: 1.38 (1.01–1.91) and 1.44 (1.06–1.95). No collinearity and no interaction between the variables of interest in initial and final models were found. No significant time-varying occupational covariate was found.

## Discussion

### Statement of principal findings

For the first time, the VISAT cohort study allowed the assessment of the links between a large panel of OC and their exposure duration and all-cause mortality, during a 20-year follow-up period. The objectives were reached by demonstrating that some OC increased the risk of all-cause mortality in the whole population, depending of exposure duration.

The exposure duration of OC was very important to consider. Indeed, exposure duration of some physical and organizational constraints such as being exposed to carrying or lifting heavy loads, loud noise, working more than 48 h/week, having an occupation requiring working in rotating shift or at night, sleeping after midnight or getting-up before 5:00 a.m. due to work increased the mortality rates and the all-cause mortality risk compared to unexposed workers. Most of them were mainly explained by modifiable factors such as cardiovascular risk factors. However, shift work without night and working at night with or without shiftwork increased significantly and independently the all-cause mortality risk. Moreover, having an occupation requiring rotating shift work and getting up before 5:00 a.m. for working increased significantly the all-cause mortality risk despite the adjustments examined. Being exposed to these OC more than 16 and 13 years, respectively in working life increased by one and a half the all-cause mortality risk.

When the potential combined effect of occupational constraints was analyzed, only the shift and night work remained significantly associated to all-cause mortality. These results highlight the need to take into account in research studies, the individual factors but also the potential counterbalanced effect of the other occupational exposures in the same working period.

### Occupational physical constraints

Concerning the impact of physical OC on all-cause mortality, the literature has provided contrasted results. Indeed, among male industrial workers, a deleterious effect of high occupational physical activity on all-cause of mortality was demonstrated in Israel Study (24), while the Belgian Physical Fitness Study did not confirm these findings (25). However, the Danish National Health Interview Surveys (26), and the Copenhagen city heart study (7) did not find any significant impact of lifting or carrying heavy loads on all-cause mortality among national samples.

Recently, a systematic review and meta-analyses found no significant association between exposure to high physical activity at work and all-cause mortality (27), in accordance with our results obtained in a general working population, after a longer follow-up (20 years) and also when others OC were considered in the same model.

The consequences of occupational noise on all-cause mortality have scarcely been explored so far. From the Copenhagen Male study (28), after a 16-year follow-up of 2,998 men, no link was found between cumulative occupational exposure to noise and all-cause mortality after multiple adjustments. However, a systematic review in 2016 (29) retained five studies that specifically investigated the link between cardiovascular mortality and occupational noise exposures. A weak association was found [HR: 1.12], encouraging deeper investigations on this issue. The VISAT study demonstrated that depending of exposure duration, being exposed to loud noise increased the risk of all-cause mortality compared to unexposed workers but these links disappeared when main cardiovascular risk factors were considered. These results stressed the need to screen cardiovascular risk factors and to deliver specific preventive advice to modify behaviors among these workers.

### Organizational occupational constraints

Organizational constraints constitute a large category of occupational risks. In 2012, a meta-analysis including prospective, retrospective and case-control studies has shown no significant effect of night-shift work on all-cause and cardiovascular mortality (11). Heterogeneity was observed between studies including several types of work patterns. However, 3 years later, another meta-analysis including only three prospective studies demonstrated a weak significant effect of night-shift work on cardiovascular death but not on all-cause mortality (10), with heterogeneity of potential confounders.

Shift work without nights and working at least 50 nights/year were independently associated with an increased risk of all-cause mortality in whole population. Our results demonstrated that being exposed to a work requiring getting up before 5:00 a.m. more than 13 years and having rotating shift work more than 16 years (at least 50 working days/year) remained significant and independently associated with all-cause mortality. Thus, the impact of work schedule depends on the number of years of exposure. These findings could be helpful for companies to implement preventive strategy by managing and limiting the exposure to these OC during the working-life of their employees. Given the large distribution of shift-night work in the labor market (15 to 20%), these findings constitute a public health and labor issue.

One of the main pathophysiological mechanisms involved, is based on circadian disturbances that are induced by shift-night work which may lead to a higher mortality risk (30).

In the Northern Ireland study (9-years of follow-up), negative consequences of long working hours (>55 h/week) on all-cause mortality have been found only in men exhibiting routine occupations while a protective effect was highlighted for male managers (6). In addition to these unexpected results and based on only one study published in 2010, a recent review of the literature did not retain any impact of long working hours on all–cause mortality (31, 32). A large meta-analysis concerning the association between long working hours and risk of coronary heart disease and stroke were conducted in 2015, including 25 studies from European, USA and Australia countries (33). The authors showed that risk of incident stroke increased significantly for workers exposed at least 48 h/week after adjustments for sex, age, socio-economic status but no association was found for cardiovascular incident events.

Our results demonstrated a negative effect of long working hours on all-cause of mortality but not after adjustment for cardiovascular risk factors and other occupational constraints. So, working long hours is probably useful for the companies ‘s efficiency, but a regular screening of cardiovascular risk among these workers needs to be implemented.

While many studies took interest in the negative effects of job strain on coronary heart disease (12), their consequences on all-cause mortality are still questioned and poorly explored. Thus, an old large US study did not find an impact of high-strain work on all-cause mortality, but an increased risk was observed for workers exposed to passive work (34). More recently and in accordance with our results, no-impact of high psychological demand and low control at work on all-cause mortality, after stratifying analyses by gender was reported in a 2016 Swedish study (35).

Starting to work before the age of 18 was significantly linked to all-cause mortality among men but the relationship disappeared when cardio-vascular risk factors were taken into account. Thus, individual factors are likely to mediate the impact of the earliness of the first job on all-cause mortality. Simply asking” “at which age did you start your first job?” during medical examination could isolate groups of increased risk of all-cause mortality and could trigger an alarm for the practitioners in order to investigate more fully the exposure to occupational constraints and classical cardiovascular risks. To our knowledge, no previous studies have investigated this topic which remains to be explored by additional studies.

### Strengths and weaknesses of the study

One of the strengths of this study was the sample size of 3,138 participants including both men and women, as a majority of studies undertaken on this topic often focused on men (5).

Because the recruitment of participants was done from routine medical visits, whatever the occupational sector, the results obtained can be generalized to a wide range of workers. The mortality rate described in this study was evaluated at 3.9 per 1,000 person-years in a population with a mean age of 45 years old. These results are consistent with those provided by previous studies, with incidence ranging from 2.6/1,000 person-years to 5.8/1,000 person-years in similar age groups and follow-up periods of 8.7 to 13 years (6, 26, 36, 37). Because data collection on exposure to OC was declarative, a reporting bias could be induced. The data for exposure duration to the job strain were not available and have limited the interpretation of findings.

A large panel of occupational constraints was considered in this study and time varying analyses were carried out. However, as in most studies focused on occupational environment, bias of “healthy worker survivor” exists. Based on development of the exposome concept (promoted by some Institutes such as the National Institute for Occupational Safety and Health), the assessment of occupational exposures over the professional carrier course (including lifestyle factors) should be promoted, including repeated measurements of occupational exposures and the links to social and territorial characteristics.

### Practical implications

#### To implement individual preventive strategy

Our findings demonstrate that some associations between OC and all-cause mortality in all population, in men and in women are mainly explained by modifiable cardiovascular risk factors such as hypertension, overweight and smoking behavior. The guideline of European society of cardiology emphasizes the need to early detect and to improve cardiovascular risk factors in general population. We can add that in clinical practice, the practitioners should take a specific care of patients who are exposed to these types of occupational constraints. Some simple questions about the OC could be included in medical visits to raise awareness by practitioners on the potential existence of cardiovascular risks among these workers.

#### To implement collective preventive strategy

Previous studies focusing on mortality took into account occupational-social categories and/or specific occupational sectors (industries, farming, offices, hospital…), but this approach cannot define what to do to improve quality of life at work.

The large number of occupational constraints tested allows determining the type of OC that need to be improved in priority by implementing preventive strategies by occupational health teams at the workplace. Concerning night-shift work, our findings emphasize the urgency to implement preventive countermeasures in workplace to reduce the deleterious effect of night-shift work on health.

## Conclusion

The links between each OC and exposure duration and all-cause mortality, and the role of individual factors were stressed but some evidence on the counterbalanced effect between the OC on mortality were also suggested. Determining which specific OC are linked to mortality is essential to implement collective preventive measures in the work place which constitute one of the important occupational health challenges of the upcoming years.

## Data availability statement

The raw data supporting the conclusions of this article will be made available by the authors, without undue reservation.

## Ethics statement

The studies involving human participants were reviewed and approved by Participants were informed and volunteered to participate in the VISAT study. Informed consent was sought and granted. The VISAT project obtained agreement from the French National Committee on Computer Files and Civil Liberties. All procedures followed international standards pertaining on human research in accordance to the Declaration of Helsinki. Acceptance by the CNIL was renewed in 2009 for the additional use of mortality data (CNIL 09.149). The patients/participants provided their written informed consent to participate in this study.

## Author contributions

YE and SH draft the work, conducted the literature review and prepared the material and methods and results and the discussion sections of text. AF helped in statistical analyses. J-CM and YE designed the study and directed its implementation in each center, including quality and control of data set. JF revised manuscript critically for important intellectual content. VB supervised statistical analyses and contributed to interpretation of data and results. CC and AF contributed to literature review and revised the manuscript for important intellectual content. All authors Agreement to be accountable for all aspects of the work in ensuring that questions related to the accuracy or integrity of any part of the work are appropriately investigated and resolved and read and approved the final manuscript.

## Funding

This work was funded by grants from the French Agence Nationale de la Recherche (ANR 2006 SEST 04 101), the Institute of Occupational Safety & Health (IOSH, UK), and the Direction Générale de la Santé (DGS), the Caisse Nationale d'Assurance Maladie des Travailleurs Salariés (CNAMTS), the Régime Social des Indépendants (RSI) and the Caisse Nationale de Solidarité pour l'Autonomie (CNSA), as part of the call for projects launched by Institut de Recherche en Santé Publique (IReSP) in 2009. All authors were independent from the funders.

## Conflict of interest

The authors declare that the research was conducted in the absence of any commercial or financial relationships that could be construed as a potential conflict of interest.

## Publisher's note

All claims expressed in this article are solely those of the authors and do not necessarily represent those of their affiliated organizations, or those of the publisher, the editors and the reviewers. Any product that may be evaluated in this article, or claim that may be made by its manufacturer, is not guaranteed or endorsed by the publisher.
