# Supplementary material for: Exposure to environmental occupational constraints and all-cause mortality: Results for men and women from a 20-year follow-up prospective cohort, the VISAT study. Be aware of shift-night workers!
Source: Front Public Health. 2022 Nov 10;10:1014517. doi: 10.3389/fpubh.2022.1014517 (PMC9687385; doi:10.3389/fpubh.2022.1014517)
Supplement: Supplementary file 1 [file Table_1.DOCX]

Supp Table A: Individual characteristics and all-cause mortality for all participants, men and women (incidence and bivariate analyses)

|  | **All N= 3138 ; death : N=242** | | | |  |
| --- | --- | --- | --- | --- | --- |
| **individual variables** | % | %  deaths | p-value | Mortality rates |  |
| **Age**  32 - 42  52  62 | 57.9  27.6  14.5 | 3.7  9.5  20.2 | **0.000** | 1.9 (1.5 to 2.4)  4.8 (3.9 to 5.9)  10.6 (8.7 to 13.1) |  |
| **Smoking status**  Never or former  Current | 68.7  (31.3 | 6.8  9.6 | **0.006** | 3.4 (2.9 to 4.1)  4.9 (4.1 to 6.1) |  |
| **Hypertension**  No  Yes | 65.9  34.0 | 4.8  13.4 | **0.000** | 3.4 (2.9 to 4.1)  6.9 (5.8 to 8.1) |  |
| **Diabetes history**  No  yes | 97.3  2.7 | 7.3  28.3 | **0.000** | 0.4 (0.3 to 0.5)  1.2 (0.7 to 1.8) |  |
| **BMI> 25kg/m²**  No  Yes | 57.9  42.1 | 5.9  10.1 | **0.000** | 2.9 (2.4 to 3.6)  5.1 ( 4.3 to 6.1) |  |
| **Other medical History**  No  Yes | 98.6  1.4 | 7.6  13.9 | 0.12 | 3.8 (3.4 to 4.3)  7.1 (3.2 to 15.6) |  |
| **Educational level**  ≤ A-level  >A-level | 41.1  58.9 | 6.2  8.8 | **0.008** | 3.1 (2.5 to 3.9)  4.4 (3.8 to 5.2) |  |

Incidence /1000 person-year and CI; p-value for Pearson’s chi2 testing associations between definite variables and all-cause mortality;
